# Are Fish and Standardized FETAX Assays Protective Enough for Amphibians? A Case Study on *Xenopus laevis* Larvae Assay with Biologically Active Substances Present in Livestock Wastes

**DOI:** 10.1100/2012/605804

**Published:** 2012-05-01

**Authors:** Federica Martini, José V. Tarazona, M. Victoria Pablos

**Affiliations:** Laboratory for Ecotoxicology, Department of Environment, Spanish National Institute for Agricultural and Food Research and Technology (INIA), Carretera de la Coruña, Km 7.5, 28040 Madrid, Spain

## Abstract

Biologically active substances could reach the aquatic compartment when livestock wastes are considered for recycling. Recently, the standardized FETAX assay has been questioned, and some researchers have considered that the risk assessment performed on fish could not be protective enough to cover amphibians. In the present study a *Xenopus laevis* acute assay was developed in order to compare the sensitivity of larvae relative to fish or FETAX assays; veterinary medicines (ivermectin, oxytetracycline, tetracycline, sulfamethoxazole, and trimethoprim) and essential metals (zinc, copper, manganese, and selenium) that may be found in livestock wastes were used for the larvae exposure. Lethal (LC_50_) and sublethal effects were estimated. Available data in both, fish and FETAX studies, were in general more protective than values found out in the current study, but not in all cases. Moreover, the presence of nonlethal effects, caused by ivermectin, zinc, and copper, suggested that several physiological mechanisms could be affected. Thus, this kind of effects should be deeply investigated. The results obtained in the present study could expand the information about micropollutants from livestock wastes on amphibians.

## 1. Introduction

Veterinary medicines are widely used to treat disease and to protect animal's health [1]. Dietary growth-enhancing feed additives (growth promoters) are also incorporated into the feed of animals to improve their growth rates [2]. One of the most important problems that could occur when livestock wastes are considered for recovery, reuse, and recycling is the presence of biologically active substances in these wastes, such as veterinary medicines, biocides, and additives for animal feed, which in small concentrations could have potential toxic effects on aquatic organisms. In the present work, five veterinary medicines and four essential metals, used as mineral supplements or food additives in livestock, have been studied in acute static tests using *Xenopus laevis* as animal model. 

The veterinary medicines selected to carry out the tests were ivermectin, oxytetracycline, tetracycline, sulfamethoxazole, and trimethoprim. The last two medicines were used maintaining the same proportions presented in the commercial chemotherapy Septrin (400 mg sulfamethoxazole and 80 mg trimethoprim). These drugs were selected because they are the most commonly used in animal husbandry within their respective categories [3–6]. The four studied essential metals were zinc (Zn), copper (Cu), manganese (Mn), and selenium (Se). Trace concentrations of essential metals are required in the diet for many biological processes, particularly enzyme functions, and they have a positive influence on livestock growth and reproduction [2]. Due to the low content of essential metals in some feeds compared to the recommendations, supplementation of these metals is necessary for most livestock species, and they are commonly added to daily rations as mineral supplements (e.g., Calfostonic, Bovis). 

For the study of acute toxicity in amphibians, the Frog Embryo Teratogenesis Assay-Xenopus (FETAX) [7] is currently used. The FETAX assay is a 4-day exposure standardized test with *Xenopus laevis* embryos from stage 8 to stage 46, according to Nieuwkoop and Faber table [8]. Over other nonstandardized tests, the FETAX assay has the advantage to evaluate a large number of parameters in one study [9]. However, since it is unknown how the exposure to toxic substances in the embryonic stage may affect the sensitivity, the results obtained from FETAX test and their use for environmental risk assessment has been questioned by Hoke and Ankley [10]. 

Thus, one of the aims of the present study is to develop an acute assay to compare potential sensitivities to toxicants between larvae and embryo in *X. laevis*. Moreover, little is known concerning the relative sensitivity of amphibians to toxicants compared with other more traditional aquatic test species, such as fish. Although there has been a substantial amount of developmental biology research with *X. laevis*, there are few toxicology data for this species compared to fish [10]. Through the present acute larvae assay, we investigated the possibility that risk assessment carried out on fish could not be protective enough for other aquatic species, such as amphibians. The results obtained could expand the existing information on ecotoxicological effects of possible micropollutants present in livestock wastes on amphibians.

## 2. Materials and Methods

### 2.1. Chemicals

Sulfamethoxazole, trimethoprim, ivermectin, and triethylene glycol (99% pure) were purchased from Sigma (Steinheim, Germany). Tetracycline (tetracycline hydrate, 99% pure) was obtained from Aldrich (Milwaukee, WI, USA). Oxytetracycline (oxytetracycline hydrochloride ≥99% pure), zinc sulphate (zinc sulphate 7-hydrate ≥99% pure) and copper chloride (copper II chloride 2-hydrate) were provided by Panreac (Barcelona, Spain). Manganese sulfate (manganese II sulfate monohydrate ≥99% pure) and sodium selenite (sodium selenite 5-hydrate for analysis) were purchased from Merck (Germany). Ultrapure water was obtained by a Milli-Q Synthesis water purification system.

### 2.2. Test Organisms

*Xenopus laevis* tadpoles, stage 47 according to* Xenopus *table of development [8], were obtained from in-house breeding of adult animals. The adults were housed in plastic aquaria in group of 10, with 40 L of dechlorinated tap water. The room temperature was set at 22 ± 1°C under a 12 : 12 h light: dark photoperiod. Frogs were fed with trout feed chopped pellets (REPRODUCTORES, Dibaq, Spain) twice a week, 2-3 h before each water change. Animal manipulation was performed in accordance with the protocol of American Society for Testing Materials [7]. Spawning of adult *X. laevis* was induced by two injections of human chorionic gonadotropin (hCG-LEPORI 2500, Angelini, Italy) into the dorsal lymph sac, spaced 8 hours apart. Male received 400 International Units (IU) of hCG at each injections. Female received 250 IU on the first injection and 800 IU on the subsequent injection. Tadpoles were changed into fresh FETAX medium with a stainless steel strainer 5 d postfertilization and fed daily on commercially available fish powder dry food (SERA MICRON, Germany) *ad libitum*.

### 2.3. Toxicity Tests

All procedures were conducted under protocols approved by the Ethics Committee for animal research of the Spanish National Institute for Agricultural and Food Research and Technology. Preliminary range-finding experiments were performed to determine the appropriate concentration ranges for the tested chemicals (data not shown). Then, short-term tests (4 d) were carried out to establish the acute lethal toxicity of tested substances and to identify potential sublethal effects. 

#### 2.3.1. Veterinary Medicines

Tests were conducted in 52 glass jars located in a water bath maintained at 22 ± 1°C on a 12 : 12 h light: dark photoperiod. Jars were placed in one 4 × 13 blocks, and treatment and replicate positions were assigned randomly. Groups of 5 larvae were exposed in each glass jar containing 100 mL of medium solutions. All tests were conducted with four replicates. Exposures took place in a reconstituted water medium suitable for Frog Embryo Teratogenesis Assay-*Xenopus*, FETAX medium [11]. Tadpoles were exposed, in a static assay, during 4 days to serial dilutions of four different drugs: S + T, TC, and OTC with initial nominal concentrations of 50 and 100 mg/L, and IVE with initial nominal concentrations of 1.075, 2.15, 4.3, 8.6, and 17.2 *μ*g/L. Because of limited aqueous solubility of tetracycline and ivermectin, triethylene glycol was used as carrier. In all experiments, the concentration of the solvent did not exceed the concentration of 1.6% (v/v), according to ASTM guidelines [7]. Larvae were checked every day for morphological abnormalities, developmental delay, abnormality swimming behaviours, and mortality, and all dead tadpoles were counted and removed.

#### 2.3.2. Essential Metals

Exposure conditions were the same as described above. Jars (*n* = 84) were randomly placed in two 3 × 14 blocks. In this case, no SC was used. Tadpoles were exposed to five geometrical serial dilutions of four different compounds: zinc sulphate (ZnSO_4_ ∗ 7 H_2_O), copper chloride (CuCl_2_ ∗ 2 H_2_O), manganese sulphate (MnSO_4_ ∗ H_2_O), and sodium selenite (NaSeO_3_ ∗ 5 H_2_O), with the aim to achieve the corresponding nominal concentrations of metals shown at Table 1.

### 2.4. Statistical Analyses

For each sample with visually distinguishable abnormalities, probit analysis (Statgraphics 5.1, StatPoint Technologies, INC., USA) was used to calculate effect concentrations in 50% of the cases (ECs_50_) with 95% confidence intervals. The same analysis was employed to calculate lethal concentrations (LCs_50_). The significance of the endpoints with respect to the control data was assessed by one way analysis of variance (ANOVA), with Fisher's least-significant difference procedure (LSD, *P* < 0.05), in the software Statgraphics 5.1.

## 3. Results

**Figure 1:**
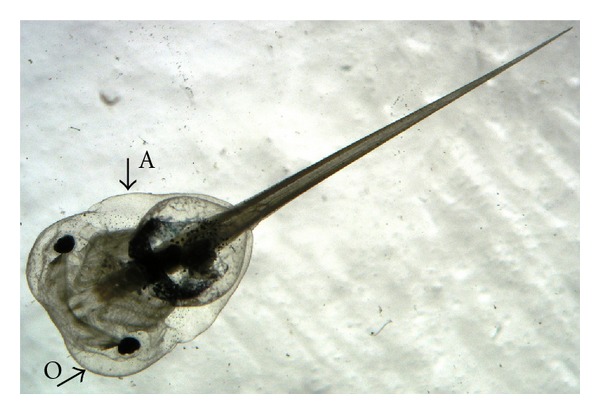
Edema in optic and abdominal areas of *X. laevis* tadpole caused by all nominal Zn exposure concentrations (2.73, 5.46, 10.91, and 21.83 mg/L), except for the lowest one (1.36 mg/L). O: optic and A: abdominal.

The embryo survival rate in the blank control (BC) and solvent control (SC) reached at least 90% throughout the duration of the tests, and SC did not show any significant effects on normal *Xenopus* development. Thus, statistical analyses were related to BC. For tadpoles treated with OTC, TC, S + T, and Mn, no lethal or sublethal effects were found; therefore, LC_50_s values were higher than the maximum exposure concentrations. For tadpoles exposed to IVE, Zn, Se, and Cu, the estimated LC_50_s and EC_50_s with their corresponding 95% confidence intervals, the sublethal effects, and the No Observed Effect Concentration (NOEC) based on sublethal effects are shown in Table 2. All tadpoles treated with IVE showed hyperactivity, rapid and uncontrollable swimming movements when a touch was given to the jar, at all tested concentrations except at the lowest one. In larvae exposed to Zn, edema (Figure 1) was detected at all exposure times and concentrations, except at 96 h at the lowest concentration. Furthermore, at 48 h the higher number of tadpoles with edema was found, while at the subsequent periods edema reabsorption in some individuals was observed (Table 2). Copper provoked two sublethal effects: developmental delay at 72 and 96 hours and abnormal pigmentation (whitish) in all tadpoles at all tested concentrations.

## 4. Discussion

The presence of xenobiotics in aquatic ecosystem does not, by itself, indicate injurious effects. Connections must be established between external levels of exposure, internal levels of tissue contamination, and early adverse effects [12]. Environmental Risk Assessment (ERA) is defined as the procedure by which the likely or actual adverse effects of pollutants and other anthropogenic activities on ecosystems and their components are estimated with a known degree of certainly using scientific methodologies [13]. Environmental Risk Assessment is currently considered the best available tool for environmental decision making [12]. The ERA of veterinary drugs for aquatic compartment uses the results of ecotoxicological tests of three basic taxa: algae (e.g., *Chlorella vulgaris*), aquatic invertebrates (e.g., *Daphnia magna*), and fish (e.g., *Oncorhynchus mykiss*). For years, the risk assessment carried out on fish was considered to be protective enough to cover other aquatic vertebrates, including amphibians. Since the mid-1990s, the significant decline suffered by amphibian populations has received the attention of both scientific community and popular media [14]. Several reasons have been put forward to explain such decline, some arising directly or indirectly from human activities, such as direct destruction of amphibian habitats by humans or chemical pollution, and others emerging from global and local climatic changes, for example, fungal and bacterial infection, which may be related to ozone depletion and an increase of ultraviolet exposure [14–16]. Due to the peculiarities of amphibians (combining aquatic and terrestrial phases in their life cycles, feeding and respiration rate, permeability of the skin), their susceptibility to contaminants in the aquatic environment could be considered greater than other aquatic organisms widely used in ecotoxicological tests, such as fish [17]. 

Environmental Risk Assessment protocols for pollutants or complex mixtures include ecotoxicological assays with fish to study effects of acute and chronic exposures on larval stages or adults. In the same way, in the case of amphibians, it would be particularly important to know the acute and chronic effects at different stages of development caused by biologically active substances such as biocides and veterinary medicines. Moreover, the lack of standardized toxicity tests with amphibians and the subsequent limitations in high-quality toxicology data for either prospective or diagnostic assessment continue to be a problem and often prevent the inclusion of amphibians in ERAs. The novel aspect of the current study lies in the use of an ecotoxicological assay with larvae stage of *X. laevis*, a not commonly used age stage, to compare the effects of acute exposures caused by biologically active substances with data obtained from FETAX or fish assays. 

Concerning sensitivity to metals and organic contaminants to facilitate their use as bioindicators of pollution stress, early-life-stage toxicity tests were used by Birge [18] to classify 25 amphibian species as very sensitive, sensitive, moderately tolerant, or tolerant in comparison with the rainbow trout (*Oncorhynchus mykiss*), a sensitive benchmark species commonly used in toxicity criterion development. *Xenopus laevis* resulted to be one of the most tolerant species. Nevertheless, in the present study, *X. laevis* was selected as animal model for amphibians, since it is cultured and handled easily in laboratory setting, and there is a relatively wide knowledge in its developmental biology [10]. Combining data for all taxa studied by Birge [18] (on Table 3 were reported the most interesting LC_50_ values), based on 573 point-to-point comparisons between amphibian and fish LC_50_ values, amphibians were more sensitive than fishes in 386 (67%) of 573 cases.Table 3 shows the differences in metal sensitivity among selected amphibian species, as well as fish. Comparisons against Table 3 and the results of the current study (Table 2) demonstrate that *X. laevis* was not always the most tolerant species. For example, *X. laevis* was more sensitive than largemouth bass (*Micropterus salmoides*) to lethal effects of Se, but was in the same range of sensitivity than goldfish (*Carassius auratus*) and rainbow trout. In the same way, X. laevis was more sensitive than fish (except for O. mykiss), and other amphibians (Ambystoma opacum and Bufo fowleri), to lethal effects of Cu. In addition, for Zn exposure, X. laevis was more sensitive than B. folweri. Moreover, considering the salt and taking into account the study of Buhl and Hamilton [19] where the 96 h LC_50_ on rainbow trout for NaSeO_3_ was 118 mg/L, the species used in the current assay was an order of magnitude more sensitive than fish to lethal effects of NaSeO_3_ (Table 2). On the contrary, for the same substance, there were no significant differences between the 96 h LC_50_ on gastrula stage of *X. laevis* (2.3 mg/L) [20] and the calculated value obtained in the present study for larvae (Table 2). In the same way, LC_50_s for OTC (>100 mg/L) and MnSO_4_ (60 mg/L) showed negligible toxicities, which were in the same range of the reported ones on rainbow trout by Office of Pesticide Programs (>116 mg/L) [21] and Davies (116 mg/L) [22], respectively. In the current study, the 48 h LC_50_ for CuCl_2_ was 2.45, while, according to data from Office of Pesticide Programs [23], on rainbow trout it was 0.01 mg/L; thus, O. *mykiss* is clearly more sensitive than *X. laevis*. Nevertheless, in comparison with FETAX assay, the toxicity of CuCl_2_ in *Xenopus *larva (Table 2) was close to the highest value of 96 h LC_50_ found by Buchwalter [24], which ranged between 0.042 and 1.180 mg/L. Published data for toxicity of ZnSO_4_ exist for a variety of fish species and amphibians. For example, Alsop and Wood [25] reported a 96 h LC_50_ value of 2.615 mg/L on rainbow trout, while in *X. laevis* blastula the 96 h LC_50_ value was 3.6 mg/L [26]. Thus, the 96 h LC_50_ of 56.44 mg/L for ZnSO_3_, obtained in the present study, showed that *X. laevis* sensitivity was lower than one order of magnitude compared with FETAX and fish assays. The ivermectin LC_50_ for *X. laevis *tadpoles was in the same range of fish and at least 100-fold less than are *Daphnia*. In fact, the 96 h LC_50_ for ivermectin on rainbow trout is 3.3 *μ*g/L and the 48 h LC_50_ value for *D. magna* is 25 ng/L [27]. Due to ivermectin mechanism of action, *Daphnia* has been determined to be the most sensitive laboratory indicator organism [27]. 

The available data about acute effects in FETAX assay are generally more protective than the values found out in the current study for *X. laevis* 47 stage larvae, but previous data derived from fish assays could not be always enough protective. For example, *X. laevis* larvae exposed to NaSeO_3_ showed a higher sensitivity than rainbow trout [19] (Table 2). In addition, the presence of no-lethal effects caused by IVE, Zn, and Cu suggested that these substances have been able to cause an organism response. For example, larvae affected by Cu were underdeveloped and colourless, while IVE impaired their locomotion and orientation. Similar effects could be problematic in natural environments by increasing the susceptibility of larvae to predation, as reported by Yuan [28] for the whitish caused by triphenyltin exposure, or reducing foraging success resulting in decreased grown and development. Changes in cognitive and psychomotor function, such as the hyperactivity induced by IVE, are commonly related to toxic neuropathy [29], while renal dysfunction, or more generally, an alter metabolism, could have caused the edema in the animals exposed to Zn (Figure 1). 

Based on the studies, FETAX assay appears to be useful in ecotoxicological hazard assessment, but fish assays might be not always protective enough for amphibian. Moreover, data from several studies indicate that late-stage amphibian larvae may be more sensitive to some chemical than traditional aquatic bioindicators [30], as occurred in the present study for metals, and for those species of amphibians that spend their entire life cycle in water (e.g., Pipidae, Cryptobranchidae), larval exposure would be more accurate than FETAX assay [18]. It is necessary to highlight the need to study and prevent amphibian species. The presence of sublethal effects caused by different compounds should be investigated considering other endpoints that may affect several physiological mechanisms in a sublethal pattern, such as immunotoxicity, or a wider range of animal larvae stages.

## Figures and Tables

**Table 1:** Nominal concentrations (mg/L) of the four salts of metal, used as water-soluble forms, and the corresponding nominal concentrations (mg/L) of the four metals considered in the current study.

Water-soluble form		Metal	
	Concentration		Concentration
Zinc sulphate 7-hydrate	6	Zinc	1.36
	12		2.73
	24		5.46
	48		10.91
	96		21.83

Copper II chloride 2-hydrate	0.6	Copper	0.22
	1.2		0.45
	2.4		0.89
	4.8		1.79
	9.6		3.58

Manganese II sulfate monohydrate	3.75	Manganese	1.22
	7.5		2.44
	15		4.88
	30		9.75
	60		19.50

Sodium selenite 5-hydrate	1.56	Selenium	0.47
	3.12		0.94
	6.25		1.88
	12.5		3.75
	25		7.51

**Table 2:** Endpoints studied at each exposure time for ivermectin (IVE), zinc (Zn), copper (Cu), selenium (Se). Lethal and effect concentrations in the 50% of the cases (EC_50_ and LC_50_) were shown at 24, 48, 72 and 96 hours. At each exposure time, the EC_50_ values were related to the corresponding sublethal effect. For the water-soluble forms of the metals: zinc sulphate (ZnSO_4_ ∗ 7 H_2_O) and sodium selenite (NaSeO_3_ ∗ 5 H_2_O) the 96 h LC_50_ were reported, and for copper chloride (CuCl_2_ ∗ 2 H_2_O) both 48 h and 96 h LC_50_s were showed.

Substance	Exposure time (hours)	LC_50_ (mg/L)	95% confidence intervals (mg/L)	EC_50_ (mg/L)	95% confidence intervals (mg/L)	NOEC^a^ (mg/L)	Sublethal effect
IVE	24	6.4 ∗ 10^−3^		>1.1 ∗ 10^−3^ <2.1 ∗ 10^−3^			hyperactivity
	48	5.6 ∗ 10^−3^	4.7 ∗ 10^−3^–6.6 ∗ 10^−3^	>1.1 ∗ 10^−3^ <2.1 ∗ 10^−3^			hyperactivity
	72	5.5 ∗ 10^−3^	4.6 ∗ 10^−3^–6.6∗10^−3^	>1.1 ∗ 10^−3^ <2.1 ∗ 10^−3^			hyperactivity
	96	5.5 ∗ 10^−3^	4.6 ∗ 10^−3^–6.6 ∗ 10^−3^	>1.1 ∗ 10^−3^ <2.1∗10^−3^		1.1 ∗ 10^−3^	hyperactivity

Zn	24	14.0	12.0–16.8	7.4	3.6–12.3		edema
	48	13.4	11.3–16.1	4.5	2.6–6.3		edema
	72	13.4	11.3–16.1	7.6	5.1–11.2		edema
	96	12.8	10.8–15.6	8.5	6.4–12.0	1.4	edema

ZnSO_4_ ∗ 7 H_2_O	96	56.4	47.4–68.7				

Cu	24	1.3	0.6–2.0	>3.6			developmental delay^b^
	48	0.9	0.3–1.3	>3.6			developmental delay
	72	0.9	0.3–1.3	0.4	0.4-0.5		developmental delay
	96	0.9	0.3–1.3	0.4	0.4-0.5	<0.2	developmental delay

CuCl_2_ ∗ 2 H_2_O	48	2.4	1–3.7				
	96	2.3	0.8–3.6				

Se	24	4.1					no detectable effect
	48	4.1					no detectable effect
	72	2.2					no detectable effect
	96	1.9					no detectable effect

NaSeO_3_ ∗ 5 H_2_O	96	6.2					

^a^Based on sublethal effects observed at 96 hours.

^b^Apart from developmental delay at 72 and 96 hours, copper provoked and abnormal pigmentation (whitish) in all tadpoles at all tested concentrations (EC_50_ < 0.22 mg/L).

**Table 3:** 96 h lethal concentration in the 50% of the cases (LC_50_, mg/L) for early-life-stage amphibians and fish exposed to copper (Cu), zinc (Zn), selenium (Se) and manganese (Mn). (Source: [18]).

Species	LC_50_			
	Cu	Zn	Se	Mn
*Rana catesbeiana*	0.02	0.08	0.07	
*Gastrophryne carolinensis*	0.02	0.01	0.09	1.42
*Rana palustris*	0.02	0.08	0.07	
*Rana pipiens*	0.05	0.05	0.14	318
*Pseudacris crucifer*	0.05			
*Ambystoma barbouri*	0.25	0.56		
*Ambystoma jeffersonianum*	0.37	1.00		
*Ambystoma texanum*	0.38	1.08		
*Ambystoma maculatum*	0.48	1.15		
*Ambystoma tigrinum*	0.50	2.00		
*Ambystoma opacum*	1.63	2.31		
*Bufo fowleri*	27.0	87.0		19.80
*Oncorhynchus mykiss*	0.09	1.06	5.17	2.91
*Micropterus salmoides*	6.68	5.18	114	25.60
*Carassius auratus*	5.20	2.52	8.91	10.40
*Ictalurus punctatus*	6.62	0.24	0.24	
